# Causal Relationships Between the Oral Microbiome and Autoimmune Diseases: A Mendelian Randomization Study

**DOI:** 10.3390/pathogens15010009

**Published:** 2025-12-20

**Authors:** Xinyu Wu, Xinye Zhang, Yuee Liang, Xuan Chen, Yuang Guo, Wanghong Zhao

**Affiliations:** 1 Department of Stomatology, Nanfang Hospital, Southern Medical University, Guangzhou 510515, China; michely_wxywxy@163.com (X.W.); xinyee_zhang@sina.com (X.Z.); yueeliang734@sina.com (Y.L.); xuan-0208@foxmail.com (X.C.); ae1371458499@foxmail.com (Y.G.); 2 Stomatological Hospital, School of Stomatology, Southern Medical University, Guangzhou 510280, China

**Keywords:** oral microbiome, autoimmune diseases, mendelian randomization, genome-wide association studies

## Abstract

The relationship between the oral microbiome and autoimmune diseases (ADs) has attracted considerable research interest. This study employed two-sample Mendelian randomization (MR) to investigate causal relationships between oral microbiota and six ADs, including rheumatoid arthritis (RA), type 1 diabetes (T1D), inflammatory bowel disease (IBD), multiple sclerosis (MS), systemic lupus erythematosus (SLE), and Sjögren’s syndrome (SS). Using genome-wide association study data from oral microbiome features and ADs, we applied inverse-variance weighted estimation complemented by sensitivity analyses and reverse MR to assess robustness and reverse causation. Analysis of 309 tongue dorsum and 285 salivary microbial features identified four tongue dorsum and five salivary taxa with genome-wide significant causal effects. Specific microbial taxa from both oral niches demonstrated protective or risk-enhancing effects for RA, T1D, IBD, and MS, while no causal associations were found for SLE or SS. These findings establish the causal role of specific oral microbiota in autoimmune pathogenesis and highlight priority candidates for further investigation as potential microbial biomarkers.

## 1. Introduction

Autoimmune diseases (ADs) are chronic disorders characterized by a loss of immunologic self-tolerance, which triggers an aberrant immune response against self-antigens, leading to sustained inflammation and tissue damage. Patients with ADs often suffer from impairment or dysfunction in tissues or organs, while also facing substantial healthcare costs [1]. This clinical and economic burden has increased due to a notable rise in the incidence of ADs over recent decades [2]. The development of ADs is driven by a complex interaction between genetic predisposition and environmental factors [3]. Recent research into the microbiome has revealed the potential role of microbial communities, especially the oral microbiota, in the onset and progression of these diseases [4]. As the entry point of both the digestive and respiratory tracts, the oral cavity hosts one of the most complex microbial ecosystems in the human body. This unique environment supports a diverse array of microorganisms that not only contribute to oral health but may also influence systemic immune responses through various mechanisms [5].

Existing studies suggest that oral dysbiosis may trigger or exacerbate autoimmune reactions in genetically susceptible individuals through mechanisms such as microbial translocation, molecular mimicry, autoantigen modification, or immune amplification [6]. In rheumatoid arthritis (RA), an increased abundance of specific pathogens, such as *Porphyromonas gingivalis* (*P. gingivalis*), has been observed in the oral cavity [7]. This bacterium produces peptidylarginine deiminase (PAD), an enzyme that catalyzes protein citrullination, disrupts immune tolerance, and can trigger the production of RA-specific autoantibodies [8]. Similarly, patients with systemic lupus erythematosus (SLE) also exhibit alterations in their oral microbiota, including changes in microbial diversity and shifts in the abundance of specific taxa [9]. Nevertheless, it remains unclear whether these microbial changes contribute to the development of ADs or merely represent consequences of the disease itself or its treatment. In many ADs, research on the oral microbiome remains in its early stages, primarily reporting associations rather than establishing causality. Therefore, clarifying the direction and nature of these relationships is crucial for identifying potential microbial biomarkers and guiding future research.

Establishing causal relationships between exposures and disease outcomes is a fundamental goal in medical and public health research. While randomized controlled trials (RCTs) are considered the gold standard in the hierarchy of evidence, they often face ethical, practical, and financial limitations [10]. Observational studies, although more feasible to conduct, are often susceptible to confounding and reverse causation, which impede reliable causal interpretation [11]. Mendelian randomization (MR) has emerged as a robust alternative methodology, rooted in Mendel’s laws of inheritance. MR uses genetic variants, primarily single-nucleotide polymorphisms (SNPs), which are randomly mixed during gamete formation and fixed at conception. Because these genetic instruments are generally independent of confounding environmental influences and are unaffected by disease processes, MR can be a powerful tool. By utilizing genetic proxies for modifiable exposures, MR approximates the randomization process of RCTs, thereby enhancing causal inference regarding the relationships between exposures and outcomes [12]. This approach has been successfully applied to explore the role of the oral microbiome in various conditions, including cancer [13,14,15,16,17], chronic obstructive pulmonary disease [18], respiratory infections [19], and epilepsy [20].

Building on this foundation, the present study employs a two-sample MR framework to explore the causal relationships between oral microbiota and six ADs: RA, type 1 diabetes (T1D), inflammatory bowel disease (IBD), multiple sclerosis (MS), SLE, and Sjögren’s syndrome (SS). This study seeks to enhance our understanding of how oral microbiota contribute to the development of ADs and aims to identify possible targets for future research.

## 2. Materials and Methods

### 2.1. Oral Microbiome Sample

The genome-wide association study (GWAS) summary statistics for the oral microbiome were obtained from the CNGBdb database, specifically derived from a recent metagenome study of the human oral microbiome, including 309 tongue dorsum microbiomes (*N* = 2017) and 285 salivary microbiomes *(N* = 1915) [21]. Stringent inclusion criteria for these samples were employed, ensuring an average sequencing depth of over 20×, a variant calling rate exceeding 98%, the absence of population stratification as assessed by principal component analysis (PCA), and the exclusion of related individuals through pairwise identity by descent. Furthermore, the study employed conservative inclusion thresholds, including an average depth of over 8×, a Hardy–Weinberg equilibrium (HWE) value greater than 10^−5^, and a genotype calling rate exceeding 98% for variants. Following rigorous quality control, the final dataset comprised 2984 individuals (2017 with tongue dorsum samples and 1915 with salivary samples). This dataset encompassed approximately 10 million common and low-frequency genetic variants, all meeting a minor allele frequency threshold of ≥0.5%.

### 2.2. Autoimmune Disease Samples

GWAS summary statistics for each of the six ADs were extracted from publicly available GWAS analyses. The GWAS data for RA were obtained from a GWAS meta-analysis, including 14,361 RA cases and 43,923 controls of European ancestry from 18 studies [22]. Data for T1D were derived from a GWAS analysis with 18,942 T1D cases and 501,638 controls of a large European cohort [23]. Summary statistics for IBD were obtained from a GWAS meta-analysis of 25,042 IBD cases and 34,915 controls of European ancestry [24]. For MS, GWAS data were acquired from a GWAS analysis of 115,803 participants, comprising 47,429 cases [25]. Additionally, the GWAS data for SLE were obtained from the Finnish Biobank, which included 213,683 European individuals, with 538 cases. Finally, SS GWAS data were also derived from the Finnish Biobank, with 214,435 European participants, 1290 of whom were cases. Detailed information on the datasets is provided in Table S1.

### 2.3. Selection of Instrumental Variables (IVs)

MR analysis requires satisfaction of three core assumptions [26]:

1. Relevance: Genetic variants serving as IVs must exhibit strong associations with exposure.
2. Independence: IVs must be independent of potential confounders.
3. Exclusion restriction: IVs should affect the outcome exclusively through the target exposure, with no direct or indirect pleiotropic pathways.

To validate these assumptions, we implemented the following IVs selection criteria for exposures (tongue dorsum microbiome, salivary microbiome) and outcomes (ADs: RA, T1D, IBD, MS, SLE, SS) [27,28]:

1. Genome-wide significance: Retained variants with *p* < 1 × 10^−5^ from exposure GWAS.
2. Instrument strength: Excluded variants with *F*-statistic ≤ 10 to mitigate weak instrument bias.
3. Minor allele frequency (MAF): Removed SNPs with MAF < 1%.
4. Linkage disequilibrium (LD) pruning: Independent SNPs were selected by performing LD-based clumping through the IEUGWAS platform, using a genomic window of 10,000 kb and an *r*^2^ threshold of 0.001.
5. Strand ambiguity: Excluded palindromic SNPs with intermediate allele frequencies (0.42 < MAF < 0.58).
6. Allele harmonization: Effect alleles were standardized across exposure and outcome datasets to ensure consistent directional effects.

### 2.4. MR Analysis

MR analysis was conducted to explore the potential causal link between oral microbiota exposure and ADs using TwoSampleMR package in R software (version 4.3.2). This analysis primarily utilized the inverse variance weighted (IVW) method [29], supplemented by Mendelian randomization-Egger (MR-Egger) [30], weighted median (WM) [31], weighted mode and simple mode methods as auxiliary tools. When the three core assumptions of MR hold, the IVW method exhibits the highest statistical efficiency [32]. In the presence of directional pleiotropy, the MR-Egger method provides relatively robust causal estimates. Meanwhile, the WM yields consistent results when up to 50% of the IVs are invalid [33,34].

To account for multiple testing, we implemented the False Discovery Rate (FDR) correction procedure to adjust *p*-values, controlling the proportion of false positives among significant findings [35]. This method requires ranking all *m* tested associations in ascending order of their *p*-values, where *k* denotes the rank of a given *p*-value. The corresponding *q*-value is computed as:

*q* = *p* × (*m*/*k*)

A significance threshold of *q* ≤ 0.10 was applied [36]. Exposure-outcome associations meeting this criterion were defined as providing suggestive evidence of causality.

### 2.5. Sensitivity Analysis

For statistically significant associations, comprehensive sensitivity analyses were conducted to verify the robustness and validity of causal inferences. Heterogeneity across instrumental variable estimates was assessed using Cochran’s *Q* test, with associations exhibiting *Q*-test *p*-values > 0.05 considered free of substantial heterogeneity [32]. Horizontal pleiotropy was evaluated through complementary approaches: MR-PRESSO analysis detected and corrected outliers in analyses with ≥3 instruments; MR-Egger regression tested directional pleiotropy (intercept term *p* > 0.05 indicating no pleiotropic bias) [34]; and for analyses limited to two SNPs (insufficient for formal pleiotropy testing), the Open Targets Genetics (https://platform.opentargets.org/, accessed on 13 May 2024) platform identified phenotypes strongly associated (*r^2^* > 0.8) with exposure-related SNPs to exclude variants with known pleiotropic effects. Furthermore, leave-one-out analysis was performed when >2 SNPs were available to evaluate disproportionate influence of individual genetic variants [37]. In order to mitigate related confounding, reverse MR analysis was conducted to assess potential reverse causation.

## 3. Results

### 3.1. IVs Selection

In accordance with the predefined selection criteria, IVs were extracted from the dataset. A uniform genome-wide significance threshold of *p* < 1 × 10^−5^ was implemented for all microbiota features. For reverse MR analyses, the primary threshold was established at *p* < 5 × 10^−8^ to ensure strict genome-wide significance. In instances yielding insufficient instruments under this stringent cutoff, the threshold was iteratively refined to *p* < 5 × 10^−6^ to derive analytically viable IVs [26]. Through this protocol, qualified IVs were systematically identified. All retained SNPs demonstrated an *F*-statistic > 10, indicating minimal susceptibility to weak instrument bias (Tables S2 and S11).

### 3.2. Causal Effects of Oral Microbiota on Six Types of ADs

**Figure 1:**
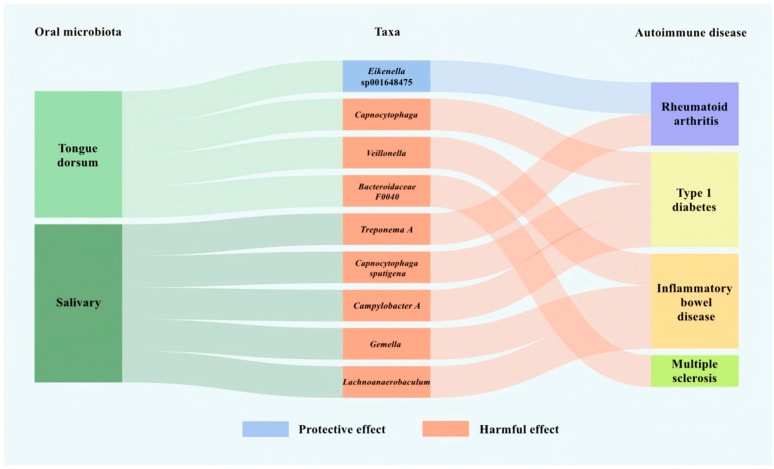
The comprehensive relationships between oral microbiota and autoimmune diseases (ADs). This Sankey diagram illustrates the associations among oral microbial niches (**left panel**), specific oral microbial taxa (**middle panel),** and ADs (**right panel**). Color coding in the diagram corresponds to the following components and association directions: Oral niches (**left panel + connecting ribbons**): dark green denotes saliva-derived oral microbiota, light green denotes tongue dorsum-derived oral microbiota; Taxa & association direction (**middle panel + connecting ribbons**): blue denotes a protective effect (the oral taxon correlates with reduced risk of the linked autoimmune disease), red denotes a harmful effect (the oral taxon correlates with increased risk of the linked autoimmune disease); ADs (**right panel**): purple denotes rheumatoid arthritis, yellow denotes type 1 diabetes, orange denotes inflammatory bowel disease, pale green denotes multiple sclerosis.

Following FDR correction to control the proportion of false positives among significant associations, our MR analyses established causal relationships between oral microbiota and ADs. Evaluation of 309 tongue dorsum features (*N* = 2017) and 285 salivary features (*N* = 1915) identified four tongue dorsum taxa and five salivary taxa with genome-wide significant causal effects on ADs (Figure 1). These associations demonstrated robustness to both heterogeneity and horizontal pleiotropy in sensitivity analyses, with no evidence of reverse causation detected in reverse MR analyses.

**Figure 2:**
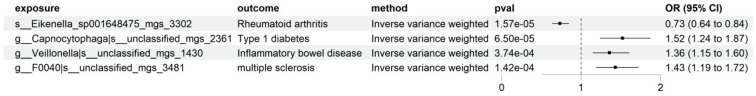
Forest plots of tongue dorsum microbiomes taxa associated with ADs (Positive results).

**Figure 3:**
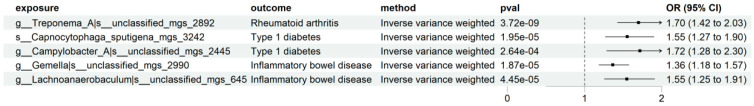
Forest plots of salivary microbiomes taxa associated with ADs (Positive results).

In tongue dorsum microbiota, *Eikenella* sp001648475 was associated with a lower risk of RA, while *Capnocytophaga*, *Veillonella*, and *Bacteroidaceae F0040* were associated with higher risks of T1D, IBD, and MS, respectively. In salivary microbiota, *Treponema A* showed a positive association with RA, while *Capnocytophaga sputigena* and *Campylobacter A* demonstrated positive causal effects on T1D, and *Gemella* and *Lachnoanaerobaculum* on IBD. Forest plots for tongue dorsum taxa associated with ADs are shown in Figure 2 and forest plots for the corresponding salivary taxa are shown in Figure 3. Detailed scatter plots and leave-one-out plots can be found in the Figures S1 and S2.

Additionally, our MR study identified several suggestive associations (Table 1), indicating potential causal links between specific oral microbiota and ADs. As phenotypic screening suggested the possible presence of horizontal pleiotropy in these associations, their interpretation should be made with caution.

### 3.3. Rheumatoid Arthritis

Our MR analysis revealed significant causal associations between specific tongue dorsum microbiota and RA. *Eikenella* sp001648475 demonstrated a protective effect against RA (IVW OR = 0.732, *p* = 1.57 × 10^−5^, FDR-*q* = 9.63 × 10^−3^, 95% CI 0.636–0.844). Sensitivity analyses confirmed robustness to heterogeneity and horizontal pleiotropy, with no evidence of reverse causality in reverse MR analysis or influential outliers in leave-one-out analysis. Meanwhile, *Leptotrichia massiliensis* was associated with lower RA risk (IVW OR = 0.618, *p* = 8.06 × 10^−5^, FDR-*q* = 2.48 × 10^−2^, 95% CI 0.487–0.785). While robust to heterogeneity and reverse causality, phenotypic screening via Open Targets Genetics revealed horizontal pleiotropy, necessitating cautious interpretation of this association.

Additionally, the salivary microbiota *Treponema A* significantly increased RA risk (IVW OR = 1.700, *p* = 3.72 × 10^−9^, FDR-*q* = 1.78 × 10^−6^, 95% CI 1.425–2.027). This association demonstrated robustness to heterogeneity and horizontal pleiotropy in sensitivity analyses, with no evidence of reverse causation (Tables S3 and S4).

### 3.4. Type 1 Diabetes

Statistically significant associations with T1D were identified in the tongue dorsum microbiota. *Capnocytophaga* exerted a measurable influence on the susceptibility of T1D (IVW OR = 1.522, *p* = 6.50 × 10^−5^, FDR-*q* = 2.23 × 10^−2^, 95% CI 1.239–1.871), with consistent sensitivity analyses. This causal relationship remained robust after rigorous assessment of heterogeneity, horizontal pleiotropy, reverse causation, and influential outliers. Conversely, *Leptotrichia massiliensis* conferred protection against T1D (IVW OR = 0.545, *p* = 3.50 × 10^−7^, FDR-*q* = 2.40 × 10^−4^, 95% CI 0.432–0.689). Sensitivity analyses indicated that the results were robust to heterogeneity but influenced by horizontal pleiotropy, as evidenced by phenotypic screening which identified traits strongly associated with the exposure SNPs. Although reverse causality was ruled out, the presence of pleiotropy necessitates cautious interpretation of the causal estimates.

The impact of salivary microbiota on T1D was also analyzed. *Capnocytophaga sputigena* elevated the risk of developing T1D (IVW OR = 1.552, *p* = 1.95 × 10^−5^, FDR-*q* = 1.08 × 10^−2^, 95% CI 1.268–1.899). Sensitivity analyses confirmed that these results were not affected by heterogeneity or horizontal pleiotropy. Reverse causality analysis indicated no reverse causal relationship, and leave-one-out analysis revealed no outliers. Additionally, *Campylobacter A* showed nominally significant risk enhancement (IVW OR = 1.718, *p* = 2.64 × 10^−4^, FDR-*q* = 7.30 × 10^−2^, 95% CI 1.285–2.298), with no evidence of heterogeneity, pleiotropy, or reverse causation (Tables S5 and S6).

### 3.5. Inflammatory Bowel Disease

We also found significant causal associations between oral microbiota and IBD. In tongue dorsum microbiota, *Veillonella* demonstrated increased IBD susceptibility (IVW OR = 1.356, *p* = 3.74 × 10^−4^, FDR-*q* = 7.42 × 10^−2^, 95% CI 1.147–1.603), with robustness to heterogeneity and horizontal pleiotropy in sensitivity analyses and no evidence of reverse causation. Conversely, *Streptococcus pseudopneumoniae* reduced IBD risk (IVW OR = 0.691, *p* = 3.11 × 10^−4^, FDR-*q* = 9.26 × 10^−2^, 95% CI 0.565–0.845). Despite robustness to heterogeneity and reverse causation, evidence of horizontal pleiotropy from phenotypic screening necessitates cautious interpretation.

Three salivary microbiota features with significant causal effects on IBD were identified. First, *Gemella* constituted a risk factor for IBD (IVW OR = 1.364, *p* = 1.87 × 10^−5^, FDR-*q* = 4.38 × 10^−3^; 95% CI 1.183–1.573). Sensitivity analyses confirmed robustness to heterogeneity, horizontal pleiotropy, and reverse causality, with no influential outliers detected in leave-one-out analysis. Second, *Lachnoanaerobaculum* elevated IBD risk (IVW OR = 1.547, *p* = 4.45 × 10^−5^, FDR-*q* = 6.94 × 10^−3^; 95% CI 1.254–1.907), demonstrating stability against heterogeneity, pleiotropic bias, and reverse causation. Third, although *Streptococcus* demonstrated a protective association with IBD (IVW OR = 0.666, *p* = 3.29 × 10^−6^, FDR-*q* = 1.54 × 10^−3^; 95% CI 0.561–0.790), phenotypic interrogation via Open Targets Genetics (https://platform.opentargets.org/, accessed on 13 May 2024) revealed horizontal pleiotropy influencing this causal estimate. While sensitivity analyses confirmed robustness to heterogeneity and reverse causation, the detected pleiotropic effects necessitate cautious interpretation of this protective relationship (Tables S7 and S8).

### 3.6. Multiple Sclerosis

The MR analysis identified significant causal relationships between specific tongue dorsum microbiota and MS. *Bacteroidaceae F0040* demonstrated a harmful association with MS (IVW OR = 1.430, *p* = 1.42 × 10^−4^, FDR-*q* = 8.29 × 10^−2^, 95% CI 1.189–1.719). Sensitivity analyses corroborated this finding and demonstrated robustness to heterogeneity and horizontal pleiotropy. Reverse causation analysis showed no evidence of bidirectional effects, and leave-one-out analysis confirmed absence of influential outliers (Tables S9 and S10).

### 3.7. Systemic Lupus Erythematosus and Sjögren’s Syndrome

However, following FDR correction with a relaxed threshold of *q* < 0.1, our MR study revealed no significant causal effects of oral microbiota, including tongue dorsum and salivary features, on SLE or SS.

## 4. Discussion

The oral microbiota, recognized as the second most complex microbial ecosystem in the human body after the gut microbiota, consists of a highly diverse community of symbiotic microorganisms, including bacteria, fungi, viruses, archaea, and protozoa [38]. With the increasing use of high-throughput sequencing technologies, research has shifted from traditional epidemiological methods to more comprehensive studies of the interactions between the oral microbiome and systemic diseases. While the relationship between the oral microbiome and ADs has been extensively investigated, the underlying mechanisms are still not fully understood. Our study utilizes MR to provide new insights into this causal association.

In the current MR analysis, due to the limited number of IVs identified under a strict significance threshold (*p* < 5 × 10^−8^), a more inclusive criterion (*p* < 1 × 10^−5^) was adopted to obtain a sufficient set of IVs, thereby enhancing the statistical power of our causal inference [26]. The strength of these candidate IVs was then rigorously evaluated using the *F*-statistic. Only IVs with an *F*-statistic > 10, confirming their robustness against weak instrument bias, were included in the final causal models. Furthermore, unlike some previous studies, our MR analysis implemented FDR correction to control the false positive rate, thereby enhancing the reliability of our findings.

RA is a systemic autoimmune disease characterized by chronic synovitis, production of autoantibodies, and progressive joint destruction. Recently, the “oral-gut-joint axis” hypothesis has garnered increased attention, proposing that the microbiota in the oral cavity and gut play a significant role in the development of RA [39]. Our MR analysis revealed a complex interaction within the oral microbiota, showcasing both risk-increasing and protective bacterial influences in RA. Notably, we discovered a protective role for the genus *Eikenella*. While *Eikenella corrodens*, a member of this genus, is typically viewed as an opportunistic pathogen found in the oral cavity and is linked to severe infections, such as septic arthritis [40], the specific species sp001648475 identified in our study exhibited a protective effect. This suggests considerable functional heterogeneity within the genus. That a member of this often pathogenic genus could confer protection against autoimmune arthritis is intriguing and implies that its mechanism may not involve simple anti-inflammatory activity, but rather a more complex ecological function. One plausible explanation is competitive exclusion [41]. *Eikenella* species on the tongue dorsum might physically occupy a niche that a more arthritogenic bacterium would otherwise colonize. Thus, its protective effect may be indirect, mediated through the maintenance of a less inflammatory overall microbial community. Conversely, the causal relationship between salivary *Treponema A* and increased RA risk is biologically plausible. As a member of the “red complex”, *Treponema denticola* is recognized as a key pathogen in periodontitis, a chronic inflammatory disease capable of triggering systemic inflammatory responses [42]. The pathogenic mechanism may operate through several pathways. Specific antigens from *Treponema* could exhibit structural similarity to host proteins, potentially inducing cross-reactive autoantibodies through molecular mimicry and thereby breaking immune tolerance [43]. Additionally, the persistent oral inflammation driven by *Treponema* could release pro-inflammatory cytokines into circulation [44], potentially exacerbating inflammatory reactions in the synovial joints of genetically susceptible individuals and contributing to the development of RA. However, one of the most striking findings of this study is the failure to confirm a causal relationship between *P. gingivalis* and RA. For decades, observational studies have considered *P. gingivalis* a key environmental factor in RA pathogenesis, primarily because it produces PAD, which converts arginine to citrulline in proteins. Antibodies against citrullinated proteins are the most specific serological markers for RA [45]. The discrepancy between our MR results and previous observational data underscores the strength of MR in disentangling causal relationships from confounded associations. Associations reported in observational studies may be influenced by confounding factors, such as smoking or immunosuppressants. Some studies have suggested that the association of *P. gingivalis* may point more toward a pre-RA autoimmune state rather than directly driving the onset of clinical arthritis [46]. Therefore, our MR results do not entirely negate the role of *P. gingivalis* in RA but instead propose a critical reinterpretation of its function. That is, it may act not as the initiator of the disease, but as an amplifier in an immune environment that is already dysregulated.

T1D is an autoimmune disorder characterized by the immune-mediated destruction of insulin-producing pancreatic β-cells. Its pathogenesis involves a combination of genetic susceptibility, environmental triggers, and immunologic dysregulation [47]. Growing evidence suggests that environmental factors, particularly infections, may play a crucial role in initiating or accelerating the autoimmune process that leads to T1D [48,49]. Our MR study provides robust causal evidence supporting the involvement of the oral microbiome in the pathogenesis of T1D. We consistently found that *Capnocytophaga* residing on the tongue dorsum and *Capnocytophaga sputigena* in saliva significantly increase the risk of T1D. This finding aligns well with the established biological characteristics of *Capnocytophaga*, a genus of Gram-negative bacteria that commonly colonizes the human oral cavity, particularly in periodontal tissues. Previous studies have reported a higher prevalence of *Capnocytophaga sputigena* and *Capnocytophaga ochracea* in the periodontal sites of children with T1D [50], and the presence of *Capnocytophaga sputigena* has been associated with gingivitis in this population [51]. Notably, *Capnocytophaga* are saccharolytic bacteria known to thrive in glucose-rich environments, such as the gingival crevicular fluid of diabetic individuals [52]. This suggests a potential vicious cycle: hyperglycemia may promote the overgrowth of *Capnocytophaga*, which in turn could exacerbate the risk of T1D through causal biological pathways. Potential mechanisms may involve bacterial components acting as superantigens or triggering molecular mimicry, which could stimulate an autoimmune response against pancreatic β-cells [53]. Moreover, our findings implicate *Campylobacter A*, a bacterial genus commonly associated with foodborne illness, as providing a plausible link between the oral microbiome and T1D. It is noteworthy that infection with *Campylobacter jejuni* represents the most frequently identified antecedent to Guillain-Barré syndrome, an autoimmune neuropathy driven by molecular mimicry between bacterial lipooligosaccharides and human nerve gangliosides [54,55]. While there is currently no direct evidence showing antigenic cross-reactivity between *Campylobacter* proteins and human islet cell antigens, the possibility of molecular mimicry remains theoretically plausible. Our MR findings provide critical causal support for this hypothesis, advancing it from a biologically plausible idea to a causally supported mechanism. These results indicate that persistent oral colonization by *Capnocytophaga* and *Campylobacter* may act as a significant environmental trigger that initiates the autoimmune cascade leading to T1D.

For IBD, our MR study identified causal roles for three oral microbial taxa in increasing disease risk, involving *Veillonella* on the tongue dorsum along with *Gemella* and *Lachnoanaerobaculum* in saliva. These results provide genetic support for the “oral-gut axis” hypothesis, which suggests that the oral cavity may act as an extra-intestinal reservoir of pro-inflammatory microbes that influence gut homeostasis. Although IBD research has traditionally focused on the gut microbiome, there is a growing recognition that the gastrointestinal tract operates as a continuous ecosystem. Oral bacteria are continually swallowed in large quantities. While most are inactivated by gastric acid, some may survive this transit and colonize the lower gut. This can potentially disrupt the indigenous microbial communities and trigger inflammatory responses [56]. Our MR findings are critical in establishing the causal direction: rather than gut inflammation secondarily altering the oral environment, the oral microbiome appears to serve as an upstream contributor to IBD pathogenesis. This conclusion is strongly reinforced by existing evidence on *Veillonella*. Studies have reported elevated abundances of *Veillonella* species in the saliva of IBD patients [57]. Furthermore, an experimental mouse model demonstrated that administration of *Veillonella parvula*, isolated from the saliva of Crohn’s disease patients, significantly worsened chemically induced colitis. The treated mice experienced greater weight loss, reduced colon length, and more severe histopathological damage compared to the controls, confirming the gut-proinflammatory potential of this oral bacterium [36]. These causal links suggest that future therapeutic strategies for IBD may need to address dysbiosis in both the gut and its upstream source in the oral cavity.

MS is a chronic autoimmune disorder that affects the central nervous system (CNS). It is characterized by immune system dysregulation, infiltration of immune cells into the CNS, demyelination, axonal damage, and ultimately neurodegeneration. Research on the role of microbes in MS has predominantly centered on the “gut–brain axis [58],” with extensive evidence indicating that gut dysbiosis can promote the migration of peripheral immune cells into the CNS and contribute to neuroinflammation [59]. However, studies examining changes in the abundance of Bacteroidaceae in the intestinal microbiota of MS patients have produced inconsistent results [60,61]. Our MR study revealed a novel causal relationship between genetically predicted elevated abundance of *Bacteroidaceae F0040* on the tongue dorsum and an increased risk of MS. This finding broadens the scope of microbial research in MS, extending from the gut to the oral cavity. Recent research on the oral microbiome in North American patients with relapsing-remitting multiple sclerosis (RRMS) has found a significant increase in the relative abundance of Bacteroidota, along with a marked elevation in the Bacteroidota/Firmicutes ratio. This finding indicates the presence of oral dysbiosis. Functional profiling of the microbiota revealed a significant enrichment of lipopolysaccharide (LPS) biosynthesis pathways in the oral microbiota of MS patients [62]. Bacteroidota are prominent producers of LPS, a powerful immunostimulant that may trigger systemic inflammatory responses, potentially leading to neuroinflammation and autoimmune pathology [63,64]. These observations support the potential causal link between Bacteroidaceae and an increased risk of MS identified in the study, suggesting that the underlying mechanism may involve systemic immune activation driven by oral dysbiosis. However, further experimental and clinical validation is still needed.

No significant evidence was found in our MR analysis, following FDR correction, to support a causal role of oral microbiota in the development of SLE or SS. This adverse finding contrasts with numerous observational studies that have consistently reported oral dysbiosis in patients with SLE or SS, including alterations in the abundance of genera such as *Streptococcus*, *Veillonella,* and *Prevotella* [65,66]. This discrepancy underscores the importance of negative MR results in clarifying causal directions. Several pathophysiological features of SLE and SS offer plausible explanations for this divergence. Both conditions involve profound systemic immune dysregulation. Additionally, SS is characterized by exocrine gland dysfunction, which leads to markedly reduced salivary flow [67]. Furthermore, immunosuppressive medications, particularly corticosteroids commonly used in treating these diseases, are known to have substantial effects on microbial communities [68]. Collectively, these factors significantly alter the oral microenvironment. It is therefore highly likely that the oral dysbiosis observed in cross-sectional studies reflects a consequence, rather than a cause, of the disease process or its treatment. This is a classic example of reverse causation, also known as confounding bias, a limitation that observational studies often struggle to overcome. In contrast, MR methods, by using genetic variants fixed at conception as IVs, are effectively resistant to such biases. However, it is worth noting that this conclusion is drawn within the limitations of the current study. The relatively more minor case numbers for SLE and SS in the available GWAS may limit our power to detect weaker causal effects, and larger genetic studies are required to rule out any causal links definitively.

The innovative aspect of this study lies in its pioneering application of MR to systematically examine the causal role of oral microbiota in the development of multiple ADs, revealing potential mechanisms by which oral microbial communities may serve as causative factors in autoimmunity. The findings not only offer new insights into the role of the oral microbiome in autoimmune disorders but also provide a theoretical foundation for future research focused on microbial modulation. For example, bacteria identified as risk factors could be prioritized as targets for subsequent mechanistic investigation and biomarker validation, while those with protective effects might be explored for their potential probiotic roles.

However, several limitations should be acknowledged. First, a relaxed significance threshold (*p* < 1 × 10^−5^) was used to obtain sufficient IVs. Although all selected instruments passed stringent *F*-statistic testing (*F* > 10) and sensitivity analyses were performed to control for weak instrument bias, this lenient criterion may theoretically retain some residual bias. Future studies utilizing GWAS data with larger sample sizes and greater statistical power may help validate our findings under more stringent significance thresholds. Second, this study is a trans-ethnic analysis, as the genetic instruments were derived from East Asian oral microbiome data, while the outcome data came from European autoimmune disease cohorts. Although the fundamental biological mechanisms of immune-microbiome interaction are likely similar across populations, differences in linkage disequilibrium patterns, allele frequencies, and gene-environment interactions may impact the applicability of the genetic instruments. Consequently, the identified SNPs need validation in ancestry-matched cohorts to confirm their consistent effects on the oral microbiome. Third, although MR employs genetic variants fixed at conception to minimize confounding, the phenotypic expression of these variants, and consequently their influence on microbiome composition, can be modulated by environmental factors such as diet, lifestyle, and medication use. These factors were not fully accounted for in the present analysis. Fourth, dynamic oral inflammatory conditions, including active periodontitis, can substantially reshape the local microbial community. This may amplify or attenuate the genetically influenced abundance of specific taxa and their associated downstream diseases. Fifth, some of the microbial features examined correspond to metagenomic species clusters whose precise taxonomic classification at the species or strain level remains unresolved. This inherent taxonomic uncertainty limits our ability to directly link the genetic signals to the specific biological functions of well-characterized pathogens, thereby constraining detailed mechanistic interpretation. Finally, as a computational epidemiological study based on summary-level genetic data, these findings are inherently hypothesis-generating and are intended to prioritize candidate microbial targets for further investigation; they do not substitute for direct causal validation through experimental models or intervention studies. In light of these considerations, the inferred causal relationships should be interpreted with caution. Future experimental and clinical studies are warranted to confirm these associations and explore their translational potential.

## 5. Conclusions

By leveraging recent GWAS data and MR analysis, this study provides genetic evidence supporting a causal role of the oral microbiome in ADs. We identified several robust causal associations between specific oral microbial taxa and autoimmune disease risk, with effects ranging from protective to detrimental. These associations remained consistent across sensitivity analyses, indicating they are promising candidates for further investigation as potential biomarkers, and point to new directions for research into oral microbiome-mediated contributions to autoimmunity.

In addition, we detected suggestive signals that may reflect plausible microbial–disease links. However, due to evidence of horizontal pleiotropy, these findings require cautious interpretation and should be regarded as hypothesis-generating. Future studies with refined genetic instruments or experimental validation are needed to clarify their potential causal roles.

## Figures and Tables

**Table 1:** Summary of causal associations and robustness assessment.

Disease	Taxonomic Label	Effect Direction	Robustness Status
Rheumatoid Arthritis (RA)	k__Bacteria|p__Proteobacteria|c__Gammaproteobacteria|o__Burkholderiales|f__Neisseriaceae|g__Eikenella|s__Eikenella_sp001648475_mgs_3302	Protective	Robust
	k__Bacteria|p__Spirochaetota|c__Spirochaetia|o__Treponematales|f__Treponemataceae|g__Treponema_A|s__unclassified_mgs_2892	Risk	Robust
	k__Bacteria|p__Fusobacteriota|c__Fusobacteriia|o__Fusobacteriales|f__Leptotrichiaceae|g__Leptotrichia|s__Leptotrichia_massiliensis_mgs_3259	Protective	Suggestive (Phenotypic screening revealed horizontal pleiotropy)
Type 1 Diabetes (T1D)	k__Bacteria|p__Bacteroidota|c__Bacteroidia|o__Flavobacteriales|f__Flavobacteriaceae|g__Capnocytophaga|s__unclassified_mgs_2361	Risk	Robust
	k__Bacteria|p__Bacteroidota|c__Bacteroidia|o__Flavobacteriales|f__Flavobacteriaceae|g__Capnocytophaga|s__Capnocytophaga_sputigena_mgs_3242	Risk	Robust
	k__Bacteria|p__Campylobacterota|c__Campylobacteria|o__Campylobacterales|f__Campylobacteraceae|g__Campylobacter_A|s__unclassified_mgs_2445	Risk	Robust
	k__Bacteria|p__Fusobacteriota|c__Fusobacteriia|o__Fusobacteriales|f__Leptotrichiaceae|g__Leptotrichia|s__Leptotrichia_massiliensis_mgs_3259	Protective	Suggestive (Phenotypic screening revealed horizontal pleiotropy)
Inflammatory Bowel Disease (IBD)	k__Bacteria|p__Firmicutes|c__Negativicutes|o__Veillonellales|f__Veillonellaceae|g__Veillonella|s__unclassified_mgs_1430	Risk	Robust
	k__Bacteria|p__Firmicutes|c__Bacilli|o__Staphylococcales|f__Gemellaceae|g__Gemella|s__unclassified_mgs_2990	Risk	Robust
	k__Bacteria|p__Firmicutes|c__Clostridia|o__Lachnospirales|f__Lachnospiraceae|g__Lachnoanaerobaculum|s__unclassified_mgs_645	Risk	Robust
	k__Bacteria|p__Firmicutes|c__Bacilli|o__Lactobacillales|f__Streptococcaceae|g__Streptococcus|s__Streptococcus_pseudopneumoniae_O_mgs_1029	Protective	Suggestive (Phenotypic screening revealed horizontal pleiotropy)
	k__Bacteria|p__Firmicutes|c__Bacilli|o__Lactobacillales|f__Streptococcaceae|g__Streptococcus|s__unclassified_mgs_880	Protective	Suggestive (Phenotypic screening revealed horizontal pleiotropy)
Multiple Sclerosis (MS)	k__Bacteria|p__Bacteroidota|c__Bacteroidia|o__Bacteroidales|f__Bacteroidaceae|g__F0040|s__unclassified_mgs_3481	Risk	Robust

Note: “Robust” indicates that the causal estimate remained significant after FDR correction and showed no evidence of heterogeneity or horizontal pleiotropy in sensitivity analyses. “Suggestive” indicates that while the main IVW estimate was significant, evidence of horizontal pleiotropy was detected, warranting cautious interpretation.

## Data Availability

All data generated or analyzed during this study are included in this published article and its Supplementary Information files.

## Supplementary Materials

The following supporting information can be downloaded at: https://www.mdpi.com/article/10.3390/pathogens15010009/s1, Figure S1: Scatter plots of tongue dorsum microbiomes taxa and salivary microbiomes taxa associated with ADs (Positive results); Figure S2: Leave-one-out plots of tongue dorsum microbiomes taxa and salivary microbiomes taxa associated with ADs; Table S1: GWAS summary statistics for the ADs included in this MR study; Table S2: The SNPs included according to selection criteria as IVs for oral microbiota and ADs in this MR study; Table S3: MR analysis results between oral microbiota and rheumatoid arthritis; Table S4: Reverse MR analysis results between oral microbiota and rheumatoid arthritis; Table S5: MR analysis results between oral microbiota and type 1 diabetes; Table S6: Reverse MR analysis results between oral microbiota and type 1 diabetes; Table S7: MR analysis results between oral microbiota and inflammatory bowel disease; Table S8: Reverse MR analysis results between oral microbiota and inflammatory bowel disease; Table S9: MR analysis results between oral microbiota and multiple sclerosis; Table S10: Reverse MR analysis results between oral microbiota and multiple sclerosis; Table S11: The SNPs included according to selection criteria as IVs in reverse MR analysis.

## References

[1] Guan S.-Y., Zheng J.-X., Feng X.-Y., Zhang S.-X., Xu S.-Z., Wang P., Pan H.-F. (2024). Global Burden Due to Modifiable Risk Factors for Autoimmune Diseases, 1990–2021: Temporal Trends and Socio-Demographic Inequalities. Autoimmun. Rev..

[2] Miller F.W. (2023). The Increasing Prevalence of Autoimmunity and Autoimmune Diseases: An Urgent Call to Action for Improved Understanding, Diagnosis, Treatment, and Prevention. Curr. Opin. Immunol..

[3] Song Y., Li J., Wu Y. (2024). Evolving Understanding of Autoimmune Mechanisms and New Therapeutic Strategies of Autoimmune Disorders. Signal Transduct. Target. Ther..

[4] Ruff W.E., Greiling T.M., Kriegel M.A. (2020). Host-Microbiota Interactions in Immune-Mediated Diseases. Nat. Rev. Microbiol..

[5] Rajasekaran J.J., Krishnamurthy H.K., Bosco J., Jayaraman V., Krishna K., Wang T., Bei K. (2024). Oral Microbiome: A Review of Its Impact on Oral and Systemic Health. Microorganisms.

[6] Baker J.L., Mark Welch J.L., Kauffman K.M., McLean J.S., He X. (2024). The Oral Microbiome: Diversity, Biogeography and Human Health. Nat. Rev. Microbiol..

[7] Cheng Z., Do T., Mankia K., Meade J., Hunt L., Clerehugh V., Speirs A., Tugnait A., Emery P., Devine D. (2021). Dysbiosis in the Oral Microbiomes of Anti-CCP Positive Individuals at Risk of Developing Rheumatoid Arthritis. Ann. Rheum. Dis..

[8] González-Febles J., Sanz M. (2021). Periodontitis and Rheumatoid Arthritis: What Have We Learned about Their Connection and Their Treatment?. Periodontol. 2000.

[9] Li B.-Z., Zhou H.-Y., Guo B., Chen W.-J., Tao J.-H., Cao N.-W., Chu X.-J., Meng X. (2020). Dysbiosis of Oral Microbiota Is Associated with Systemic Lupus Erythematosus. Arch. Oral Biol..

[10] Shiely F., Shea N.O., Murphy E., Eustace J. (2024). Registry-Based Randomised Controlled Trials: Conduct, Advantages and Challenges—A Systematic Review. Trials.

[11] Fu R., Kim S.J. (2021). Inferring Causality from Observational Studies: The Role of Instrumental Variable Analysis. Kidney Int..

[12] Smith G.D., Ebrahim S. (2003). “Mendelian Randomization”: Can Genetic Epidemiology Contribute to Understanding Environmental Determinants of Disease?. Int. J. Epidemiol..

[13] Feng K., Ren F., Wang X. (2023). Association between Oral Microbiome and Seven Types of Cancers in East Asian Population: A Two-Sample Mendelian Randomization Analysis. Front. Mol. Biosci..

[14] Feng K., Ren F., Shang Q., Wang X., Wang X. (2024). Association between Oral Microbiome and Breast Cancer in the East Asian Population: A Mendelian Randomization and Case–Control Study. Thorac. Cancer.

[15] Hu K., Huang T., Zhang Y., Ye Z., Guo J., Zhou H. (2024). A Causal Association between Esophageal Cancer and the Oral Microbiome: A Mendelian Randomization Study Based on an Asian Population. Front. Cell. Infect. Microbiol..

[16] Gu Y., Jiang L., Shui M., Luo H., Zhou X., Zhang S., Jiang C., Huang J., Chen H., Tang J. (2024). Revealing the Association between East Asian Oral Microbiome and Colorectal Cancer through Mendelian Randomization and Multi-Omics Analysis. Front. Cell. Infect. Microbiol..

[17] Wu Z., Peng Q., Ren Z., Xu X., Jiang X., Yang W., Han Y., Oyang L., Lin J., Peng M. (2025). Causal Association between Oral Microbiota and Oral Cancer: A Mendelian Randomization Study. Sci. Rep..

[18] Zhou X., Wen F., Qiu H., Li J. (2025). Genetic Evidence from Mendelian Randomization Strengthens the Causality between Oral Microbiome and Chronic Obstructive Pulmonary Disease. Medicine.

[19] He J., Mao N., Lyu W., Zhou S., Zhang Y., Liu Z., Xu Z. (2024). Association between Oral Microbiome and Five Types of Respiratory Infections: A Two-Sample Mendelian Randomization Study in East Asian Population. Front. Microbiol..

[20] Zhao C., Chen F., Li Q., Zhang W., Peng L., Yue C. (2024). Causal Relationship between Oral Microbiota and Epilepsy Risk: Evidence from Mendelian Randomization Analysis in East Asians. Epilepsia Open.

[21] Liu X., Tong X., Zhu J., Tian L., Jie Z., Zou Y., Lin X., Liang H., Li W., Ju Y. (2021). Metagenome-Genome-Wide Association Studies Reveal Human Genetic Impact on the Oral Microbiome. Cell Discov..

[22] Okada Y., Wu D., Trynka G., Raj T., Terao C., Ikari K., Kochi Y., Ohmura K., Suzuki A., Yoshida S. (2014). Genetics of Rheumatoid Arthritis Contributes to Biology and Drug Discovery. Nature.

[23] Chiou J., Geusz R.J., Okino M.-L., Han J.Y., Miller M., Melton R., Beebe E., Benaglio P., Huang S., Korgaonkar K. (2021). Interpreting Type 1 Diabetes Risk with Genetics and Single-Cell Epigenomics. Nature.

[24] de Lange K.M., Moutsianas L., Lee J.C., Lamb C.A., Luo Y., Kennedy N.A., Jostins L., Rice D.L., Gutierrez-Achury J., Ji S.-G. (2017). Genome-Wide Association Study Implicates Immune Activation of Multiple Integrin Genes in Inflammatory Bowel Disease. Nat. Genet..

[25] International Multiple Sclerosis Genetics Consortium (2019). Multiple Sclerosis Genomic Map Implicates Peripheral Immune Cells and Microglia in Susceptibility. Science.

[26] Burgess S., Davey Smith G., Davies N.M., Dudbridge F., Gill D., Glymour M.M., Hartwig F.P., Kutalik Z., Holmes M.V., Minelli C. (2023). Guidelines for Performing Mendelian Randomization Investigations: Update for Summer 2023. Wellcome Open Res..

[27] Sanderson E., Spiller W., Bowden J. (2021). Testing and Correcting for Weak and Pleiotropic Instruments in Two-Sample Multivariable Mendelian Randomization. Stat. Med..

[28] Auton A., Brooks L.D., Durbin R.M., Garrison E.P., Kang H.M., Korbel J.O., Marchini J.L., McCarthy S., McVean G.A., 1000 Genomes Project Consortium (2015). A Global Reference for Human Genetic Variation. Nature.

[29] Burgess S., Foley C.N., Zuber V. (2018). Inferring Causal Relationships Between Risk Factors and Outcomes from Genome-Wide Association Study Data. Annu. Rev. Genom. Hum. Genet..

[30] Burgess S., Thompson S.G. (2017). Interpreting Findings from Mendelian Randomization Using the MR-Egger Method. Eur. J. Epidemiol..

[31] Bowden J., Davey Smith G., Haycock P.C., Burgess S. (2016). Consistent Estimation in Mendelian Randomization with Some Invalid Instruments Using a Weighted Median Estimator. Genet. Epidemiol..

[32] Hemani G., Zheng J., Elsworth B., Wade K.H., Haberland V., Baird D., Laurin C., Burgess S., Bowden J., Langdon R. (2018). The MR-Base Platform Supports Systematic Causal Inference across the Human Phenome. Elife.

[33] Yavorska O.O., Burgess S. (2017). MendelianRandomization: An R Package for Performing Mendelian Randomization Analyses Using Summarized Data. Int. J. Epidemiol..

[34] Verbanck M., Chen C.-Y., Neale B., Do R. (2018). Detection of Widespread Horizontal Pleiotropy in Causal Relationships Inferred from Mendelian Randomization between Complex Traits and Diseases. Nat. Genet..

[35] Benjamini Y., Hochberg Y. (1995). Controlling the False Discovery Rate: A Practical and Powerful Approach to Multiple Testing. J. R. Stat. Soc. Ser. B Stat. Methodol..

[36] Xiang B., Hu J., Zhi M., Xu Z., Zhang M., Peng X. (2025). P0213 Salivary Veillonella Parvula from Crohn’s Disease Patients Exacerbates Intestinal Inflammation. J. Crohn’s Colitis.

[37] Bowden J., Del Greco M.F., Minelli C., Zhao Q., Lawlor D.A., Sheehan N.A., Thompson J., Davey Smith G. (2019). Improving the Accuracy of Two-Sample Summary-Data Mendelian Randomization: Moving beyond the NOME Assumption. Int. J. Epidemiol..

[38] Caselli E., Fabbri C., D’Accolti M., Soffritti I., Bassi C., Mazzacane S., Franchi M. (2020). Defining the Oral Microbiome by Whole-Genome Sequencing and Resistome Analysis: The Complexity of the Healthy Picture. BMC Microbiol..

[39] Drago L., Zuccotti G.V., Romanò C.L., Goswami K., Villafañe J.H., Mattina R., Parvizi J. (2019). Oral-Gut Microbiota and Arthritis: Is There an Evidence-Based Axis?. J. Clin. Med..

[40] *Eikenella corrodens* in a Patient with Septic Arthritis: A Case Report—PubMed. https://pubmed.ncbi.nlm.nih.gov/40270686/.

[41] Louca S., Doebeli M. (2016). Transient Dynamics of Competitive Exclusion in Microbial Communities. Environ. Microbiol..

[42] Holt S.C., Ebersole J.L. (2005). *Porphyromonas gingivalis*, *Treponema denticola*, and *Tannerella forsythia*: The “Red Complex”, a Prototype Polybacterial Pathogenic Consortium in Periodontitis. Periodontol. 2000.

[43] Lucchese A. (2019). Periodontal Bacteria and the Rheumatoid Arthritis-Related Antigen RA-A47: The Cross-Reactivity Potential. Curr. Opin. Rheumatol..

[44] Santos W.S., Solon I.G., Branco L.G.S. (2025). Impact of Periodontal Lipopolysaccharides on Systemic Health: Mechanisms, Clinical Implications, and Future Directions. Mol. Oral Microbiol..

[45] Perricone C., Ceccarelli F., Matteo S., Di Carlo G., Bogdanos D.P., Lucchetti R., Pilloni A., Valesini G., Polimeni A., Conti F. (2019). *Porphyromonas gingivalis* and Rheumatoid Arthritis. Curr. Opin. Rheumatol..

[46] De Vries C., Cîrciumaru A., Mathsson-Alm L., Westerlind H., Dehara M., Kisten Y., Potempa B., Potempa J., Hensvold A., Lundberg K. (2025). *Porphyromonas gingivalis* Associates with the Presence of Anti-Citrullinated Protein Antibodies, but Not with the Onset of Arthritis: Studies in an at-Risk Population. RMD Open.

[47] Ilonen J., Lempainen J., Veijola R. (2019). The Heterogeneous Pathogenesis of Type 1 Diabetes Mellitus. Nat. Rev. Endocrinol..

[48] Wen L., Ley R.E., Volchkov P.Y., Stranges P.B., Avanesyan L., Stonebraker A.C., Hu C., Wong F.S., Szot G.L., Bluestone J.A. (2008). Innate Immunity and Intestinal Microbiota in the Development of Type 1 Diabetes. Nature.

[49] Kostic A.D., Gevers D., Siljander H., Vatanen T., Hyötyläinen T., Hämäläinen A.-M., Peet A., Tillmann V., Pöhö P., Mattila I. (2015). The Dynamics of the Human Infant Gut Microbiome in Development and in Progression toward Type 1 Diabetes. Cell Host Microbe.

[50] Wang L., Gong C., Wang R., Wang J., Yang Z., Wang X. (2024). A Pilot Study on the Characterization and Correlation of Oropharyngeal and Intestinal Microbiota in Children with Type 1 Diabetes Mellitus. Front. Pediatr..

[51] Duque C., João M.F.D., Camargo G.A.d.C.G., Teixeira G.S., Machado T.S., Azevedo R.d.S., Mariano F.S., Colombo N.H., Vizoto N.L., Mattos-Graner R.d.O. (2017). Microbiological, Lipid and Immunological Profiles in Children with Gingivitis and Type 1 Diabetes Mellitus. J. Appl. Oral Sci..

[52] Ciantar M., Gilthorpe M.S., Hurel S.J., Newman H.N., Wilson M., Spratt D.A. (2005). *Capnocytophaga* Spp. in Periodontitis Patients Manifesting Diabetes Mellitus. J. Periodontol..

[53] Pahari S., Chatterjee D., Negi S., Kaur J., Singh B., Agrewala J.N. (2017). Morbid Sequences Suggest Molecular Mimicry between Microbial Peptides and Self-Antigens: A Possibility of Inciting Autoimmunity. Front. Microbiol..

[54] Latov N. (2022). *Campylobacter jejuni* Infection, Anti-Ganglioside Antibodies, and Neuropathy. Microorganisms.

[55] Nyati K.K., Nyati R. (2013). Role of *Campylobacter jejuni* Infection in the Pathogenesis of Guillain-Barré Syndrome: An Update. Biomed Res. Int..

[56] Kunath B.J., De Rudder C., Laczny C.C., Letellier E., Wilmes P. (2024). The Oral–Gut Microbiome Axis in Health and Disease. Nat. Rev. Microbiol..

[57] Hammad M.I., Conrads G., Abdelbary M.M.H. (2023). Isolation, Identification, and Significance of Salivary *Veillonella* Spp., *Prevotella* Spp., and *Prevotella salivae* in Patients with Inflammatory Bowel Disease. Front. Cell. Infect. Microbiol..

[58] Adamczyk-Sowa M., Medrek A., Madej P., Michlicka W., Dobrakowski P. (2017). Does the Gut Microbiota Influence Immunity and Inflammation in Multiple Sclerosis Pathophysiology?. J Immunol Res.

[59] Yadav S.K., Ito K., Dhib-Jalbut S. (2023). Interaction of the Gut Microbiome and Immunity in Multiple Sclerosis: Impact of Diet and Immune Therapy. Int. J. Mol. Sci..

[60] Zhang X., Wei Z., Liu Z., Yang W., Huai Y. (2024). Changes in Gut Microbiota in Patients with Multiple Sclerosis Based on 16s rRNA Gene Sequencing Technology: A Review and Meta-Analysis. J. Integr. Neurosci..

[61] Zhu S., Jiang Y., Xu K., Cui M., Ye W., Zhao G., Jin L., Chen X. (2020). The Progress of Gut Microbiome Research Related to Brain Disorders. J. Neuroinflamm..

[62] Ganesan S.M., Yadav M., Ghimire S., Lehman P.C., Patel A.J., Woods S., Olalde H., Hoang J., Paullus M., Cherwin C. (2025). Relapsing–Remitting Multiple Sclerosis Is Associated With a Dysbiotic Oral Microbiome. Ann. Clin. Transl. Neurol..

[63] Lukiw W.J. (2016). *Bacteroides fragilis* Lipopolysaccharide and Inflammatory Signaling in Alzheimer’s Disease. Front. Microbiol..

[64] Wu Z., Guo J., Zhang Z., Gao S., Huang M., Wang Y., Zhang Y., Li Q., Li J. (2024). Bacteroidetes Promotes Esophageal Squamous Carcinoma Invasion and Metastasis through LPS-Mediated TLR4/Myd88/NF-κB Pathway and Inflammatory Changes. Sci. Rep..

[65] Rusthen S., Kristoffersen A.K., Young A., Galtung H.K., Petrovski B.É., Palm Ø., Enersen M., Jensen J.L. (2019). Dysbiotic Salivary Microbiota in Dry Mouth and Primary Sjögren’s Syndrome Patients. PLoS ONE.

[66] Guo J., Cui G., Huang W., Zheng Z., Li T., Gao G., Huang Z., Zhan Y., Ding S., Liu S. (2023). Alterations in the Human Oral Microbiota in Systemic Lupus Erythematosus. J. Transl. Med..

[67] Muszyński D., Kucharski R., Marek-Trzonkowska N., Kalinowska M., Brzóska A., Bolcewicz M., Kalinowski L., Kaźmierczak-Siedlecka K. (2025). Treatment of Xerostomia in Sjögren’s Syndrome—What Effect Does It Have on the Oral Microbiome?. Front. Cell. Infect. Microbiol..

[68] Gao L., Cheng Z., Zhu F., Bi C., Shi Q., Chen X. (2022). The Oral Microbiome and Its Role in Systemic Autoimmune Diseases: A Systematic Review of Big Data Analysis. Front. Big Data.

