# Supplementary material for: Causal Relationships Between the Oral Microbiome and Autoimmune Diseases: A Mendelian Randomization Study
Source: Pathogens. 2025 Dec 20;15(1):9. doi: 10.3390/pathogens15010009 (PMC12845399; doi:10.3390/pathogens15010009)
Supplement: Supplementary file 1 [file pathogens-15-00009-s001.zip › Figures S1 and S2.pdf]

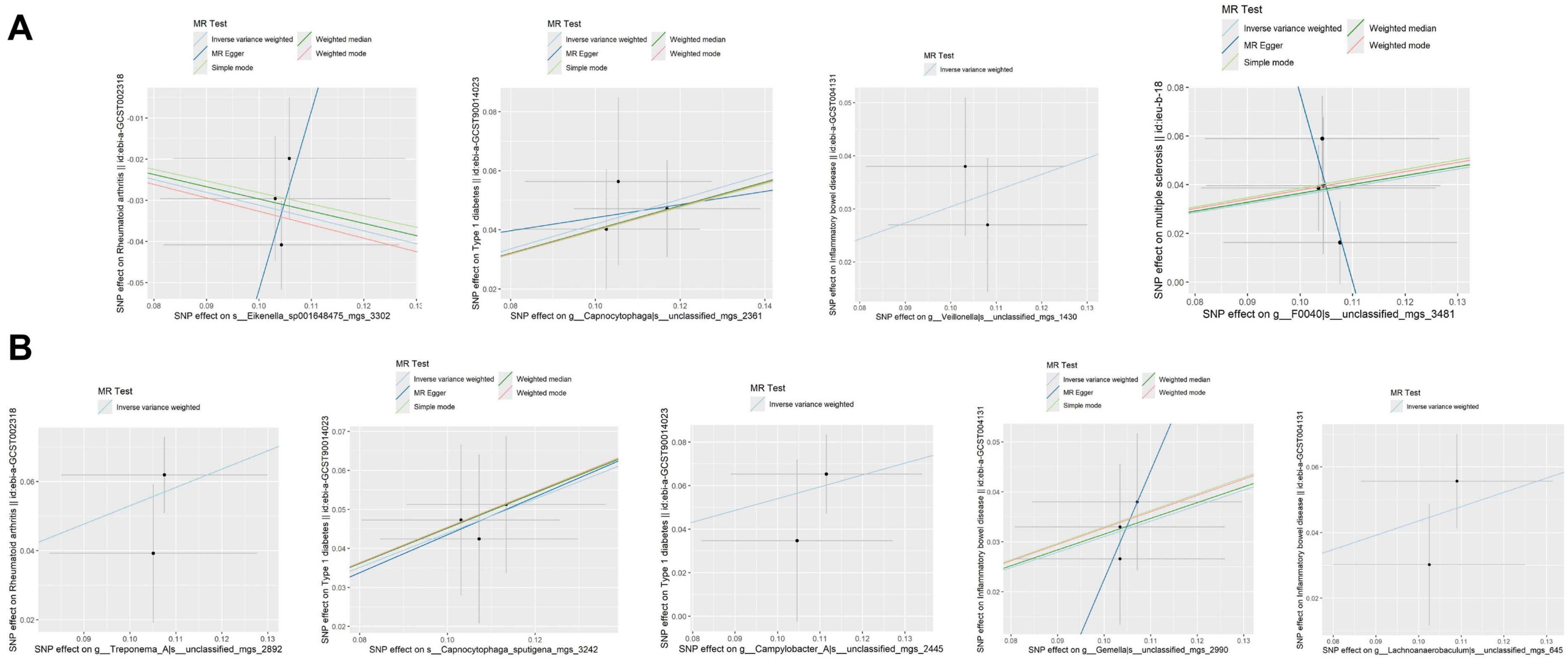

Figure S1. Scatter plots of tongue dorsum microbiomes taxa (A) and salivary microbiomes taxa (B) associated with ADs (Positive results). Each black circle represents an individual SNP used as an instrumental variable in the MR analysis.

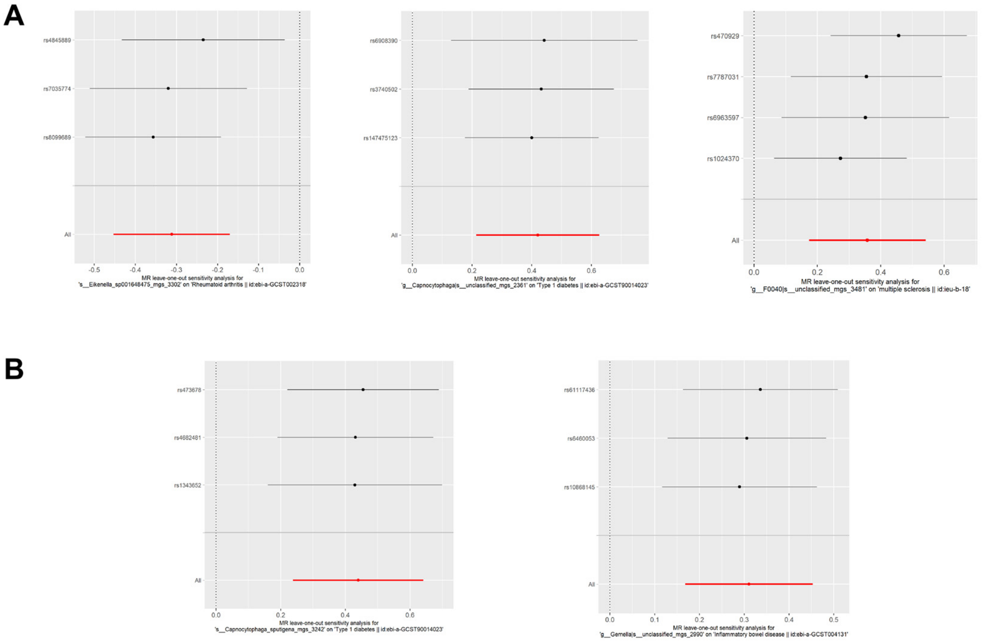

Figure S2. Leave-one-out plots of tongue dorsum microbiomes taxa (A) and salivary microbiomes taxa (B) associated with ADs.
